# Low Protective Efficacy of the Current Japanese Encephalitis Vaccine against the Emerging Genotype 5 Japanese Encephalitis Virus

**DOI:** 10.1371/journal.pntd.0004686

**Published:** 2016-05-03

**Authors:** Lei Cao, Shihong Fu, Xiaoyan Gao, Minghua Li, Shiheng Cui, Xiaolong Li, Yuxi Cao, Wenwen Lei, Zhi Lu, Ying He, Huanyu Wang, Jinghua Yan, George Fu Gao, Guodong Liang

**Affiliations:** 1 State Key Laboratory of Infectious Disease Prevention and Control, National Institute for Viral Disease Control and Prevention, Chinese Center for Disease Control and Prevention, Beijing, People’s Republic of China; 2 Collaborative Innovation Center for Diagnosis and Treatment of Infectious Diseases, Hangzhou, People’s Republic of China; 3 CAS Key Laboratory of Pathogenic Microbiology and Immunology, Institute of Microbiology, Chinese Academy of Sciences, Beijing, People’s Republic of China; The George Washington University School of Medicine and Health Sciences, UNITED STATES

## Abstract

**Background:**

The current Japanese encephalitis (JE) vaccine derived from G3 JE virus (JEV) can induce protective immunity against G1–G4 JEV genotypes. However, protective efficacy against the emerging G5 genotype has not been reported.

**Methods/Principal Findings:**

Using *in vitro* and *in vivo* tests, biological phenotype and cross-immunoreactions were compared between G3 JEV and G5 JEV (wild strains). The PRNT_90_ method was used to detect neutralizing antibodies against different genotypes of JEV in JE vaccine-immunized subjects and JE patients. In JE vaccine-immunized mice, the lethal challenge protection rates against G3 and G5 JEV wild strains were 100% and 50%, respectively. The seroconversion rates (SCRs) of virus antibodies against G3 and G5 JEV among vaccinated healthy subjects were 100% and 35%, respectively. All clinically identified JE patients showed high levels of G3 JEV neutralizing antibodies (≥1:10–1280) with positive serum geometric mean titers (GMTs) of 43.2, while for G5 JEV, neutralizing antibody conversion rates were only 64% with positive serum GMTs of 11.14. Moreover, the positive rate of JEV neutralizing antibodies against G5 JEV in pediatric patients was lower than in adults.

**Conclusions/Significance:**

Low levels of neutralizing/protective antibodies induced by the current JE vaccine, based on the G3 genotype, were observed against the emerging G5 JEV genotype. Our results demonstrate the need for more detailed studies to reevaluate whether or not the apparent emergence of G5 JEV can be attributed to failure of the current vaccine to induce appropriate immune protectivity against this genotype of JEV.

## Author Summary

The human disease Japanese encephalitis (JE) can be prevented by vaccination, although it is not entirely clear if the emerging JEV G5 genotype can be controlled using the vaccine based on the G3 genotype. Consequently, we systematically compared G3 and G5 cross-neutralizing immune responses in vaccinated humans and, separately, cross-protective immune responses in mice using the current G3 JE vaccine to induce the immunity. Based on these results, we propose that the current JE vaccine derived from G3 JE virus (JEV) does not provide adequate levels of protection against the emerging G5 JEV genotype.

## Introduction

Japanese encephalitis (JE), probably the world’s most frequently occurring viral encephalitis, is a neurological infectious disease caused by Japanese encephalitis virus (JEV), transmitted via mosquito bite [1]. The mortality rate of JE patients is 30–35% and 50% of JE survivors live with neurological sequelae. Therefore, JE is considered a disease with significant public health and economic burdens [2, 3]. JE, prevalent in 24 Asia-Pacific countries, is a mosquito-borne zoonotic natural focal disease with an estimated 67,900 cases each year. Approximately, 30 million people live in JE-endemic areas [4]. In addition, with increased international travel and business to these areas, more people are at risk of JE infection [5, 6], presenting a potentially serious international public health problem.

Since there is no effective therapeutic antiviral treatment, JE vaccination is the most effective prevention and control measure [7]. Studies have shown that the current vaccine derived from the G3 JEV can protect against G1–G4 JEV infections [8]. However, G5 JEV, first discovered in 1951, has not been epidemic over the last 60 years but reemerged in 2009 and simultaneously occurred on the Asian continent in East Asia (Korea) and southern Asia (Tibet) [9, 10, 11], presenting new challenges for JE prevention and control. Importantly, G5 JEV and G1–G4 JEV differ significantly in their molecular biological characteristics [12, 13, 14]. Nevertheless, a recent report demonstrated protective effects of the current JE vaccine against G5 JEV in animals [14]. However, whether or not humans immunized with the current G3 JEV-based vaccine can produce protective antibodies against the emerging G5 JEV is currently unknown. Moreover, it is not known if JE-infected patients have protective antibodies against G5 JEV.

Here, using wild-type strains of G3 and G5 JEV genotypes, we report comparative studies of neutralizing antibody levels against G5 JEV in both JE-vaccinated subjects and clinically diagnosed JE patients.

## Methods

### Cell lines and cell culture

BHK-21 cells (newborn hamster kidney cells) were maintained at 37°C with 5% CO_2_ and cultured in minimum essential medium (MEM) (11095–080, GIBCOTM, Invitrogen, USA) supplemented with 10% fetal bovine serum (FBS) (10099–141, GIBCOTM, Invitrogen, Australia) and Penicillin (1000 unit/mL)-Streptomycin (100 μg/mL; PS) (15070–063, GIBCOTM, Invitrogen, USA). JEV-infected BHK cells were cultured in MEM containing 2% FBS and PS.

### Virus

All JEV strains used in this study were isolated from field samples using cell culture and stored at low passage level in our laboratory: Department of Viral Encephalitis and Arbovirus, Institute for Viral Disease Control and Prevention, Chinese Center for Disease Control and Prevention (IVDC, China CDC). G1 JEV was GZ56 strain (GenBank: HM366552) [15], G3 JEV was P3 strain (GenBank: JEU47032) [16], and G5 JEV was XZ0934 strain (GenBank: JF915894.1) [9].

### Measurement of virus multiplication (plaque assay)

BHK-21 cells were inoculated at a multiplicity of infection (MOI) of 0.01 and the cytopathic effects (CPE) of the cells were observed. Simultaneously, aliquots of the infected media were collected every 12 h and measured using plaque assays to generate a viral reproduction curve [14]. Aliquots of 10-fold diluted virus suspension (from 10^−1^ to 10^−6^) were added to BHK-21 cells in 6-well plates (0.1 mL/well). After 1 h of adsorption at 37°C, the cells were overlaid with 1.3% methylcellulose-MEM medium containing 2% FBS (5 mL/well). After culturing for 3 ~ 5 days, the cells were stained with crystal violet and virus titres expressed as plaque-forming units (pfu) were calculated. For each virus, the diameter of 8 plaques was measured and the mean plaque size in mm and standard error (SEM) was calculated.

### Serum samples

Serum samples from JE-vaccinated children (3 mL of blood) were collected from two-year-old children 28 days before and after vaccination SA14-14-2 JE live attenuated vaccine (LAV), (Wuhan Institute of Biological Products Co. Ltd Liability, Wuhan, China). Serum samples (5 mL of blood) were collected from patients diagnosed with JE. All serum samples were obtained from whole blood after clotting at room temperature and centrifuged at 5,000 rpm for 5 min. Aliquots of serum were stored at -80°C until use.

### Ethics statement

The implementation agencies of the present study were the National Reference Laboratory of Japanese Encephalitis and the World Health Organization JE Regional Reference Laboratory, China (WHO-JE-RRL, China). Therefore, all the human serum samples used in this study were from those collected and maintained during the JE epidemiological monitoring period by these JE reference laboratories. All adult subjects provided written informed consent, and a parent or guardian of any child participant provided informed consent on their behalf. This research project was approved by the Ethics Committee of the IVDC, China CDC (No.2014112509).

### Detection of IgM antibodies against JEV

Two kits were used to duplicate test serum samples collected from JE patients: the JE-Dengue IgM Combo ELISA (PanBio, Brisbane, Australia), and the JEV IgM-Capture ELISA kit (Shanghai B & C Enterprise Development Co. Ltd, Shanghai, People’s Republic of China). Methods and result interpretation were performed according to manufacturers’ instructions [17]. Both detection kits were Capture ELISA.

### Animal experiments

BALB/c mice were purchased from Beijing Vital River Laboratory Animal Technology Co., Ltd. (quality qualifier SYXK Beijing 2012–0022) and were female except for the neonatal mice. All mice were housed under pathogen-free conditions at the China CDC animal facility. All animal experiments were conducted in strict compliance with the regulations set by the Animal Ethics Committee of China CDC (No.2014112509).

#### Lethality of viruses to mice

BALB/c neonatal mice (1–2 days old, 12 mice/group) were intracerebrally (i.c.) inoculated with 1×10^5^ pfu/mL JEV (0.01 mL/each); 5 ~ 6-week-old BALB/c (5 mice/group) were intraperitoneally (i.p.) inoculated with 10-fold diluted JEV (from 10^−1^~10^−6^, 0.03 mL/each), followed by a sham i.c. injection. The incidence and mortality of mice were observed and recorded every day and the Reed-Muench method was used to calculate the 50% lethal dose (LD_50_) of JEV to adult mice.

#### Viral immunology

G3 and G5 JEV (2000 pfu/300 μL each) were thermally inactivated at 56°C for 30 min and then 4-week-old BALB/c mice were separately immunized (7 mice/group) by multiple subcutaneous injections (300 μL/each) with an equal volume of mixed Freund’s complete adjuvant at day 0 followed by direct i.p. injection (300 μL/each) every two weeks for a total of three times. Two weeks after the third immunization, sera were collected to test the cross-immunoreaction between G3 and G5 JEV.

#### Immunity protection of lethal challenge experiments in mice

The two vaccines used for immunization of mice were SA14-14-2 live attenuated JE vaccine (LAV) (201311103–1, Wuhan Institute of Biological Products Co., Ltd., Wuhan, China) and P3 lyophilized JE inactivated purified vaccine (IPV) (201302B15, Liaoning Chengda Bio Co., Ltd., Liaoning, China). Animals were vaccinated i.p. with either a single dose of 10^−2^ (2510 pfu), 10^−3^ (251 pfu) or 10^−4^ (25 pfu) LAV or with 2 i.p. doses of the IPV (1:5, 1:25 or 1:125 dilution of stock vaccine) 7 days apart. Fourteen days after the initial immunization, mice were challenged i.p. with a dose containing an estimated 500 LD_50_ of the G3 or G5 JEV strain (0.3 mL/each). The mice were monitored frequently mortality rates were recorded until 21 days post-challenge [18].

### Neutralization tests

Serum neutralizing antibody to JEV was detected using the plaque reduction neutralization test (PRNT) method. The inactivated serum was diluted two-fold (from 1:5 to 1:1280) and mixed with an equal volume of 200 pfu JEV, incubated in 37°C for 1 h. The mixture was then added to 6-well BHK-21 plates for 1 h followed by overlaying with 1.3% methylcellulose-MEM medium containing 2% FBS for 3 ~ 5 days. The plaques were stained with crystal violet and counted. Neutralizing antibody titers were calculated as the reciprocal of the highest dilution resulting in 90% plaque reduction (PRNT_90_) compared to an unvaccinated control serum [19].

### Statistical analyses

In this study, the positive cut-off value of neutralizing antibody titer was defined as PRNT_90_ ≧1:10. Seropositive subjects were defined as those with a titer above or equal to the cut-off value and when negative, the result was given an arbitrary value of 5 for calculating the geometric mean titer (GMT). All data were processed and drawn using GraphPad Prism 5.0 software (GraphPad, La Jolla, CA, USA), Student’s *t*-tests were used for all analyses and a P-value <0.05 was considered statistically significant.

## Results

### General phenotypic characteristics of G5 and G3 JEV

**Fig 1:**
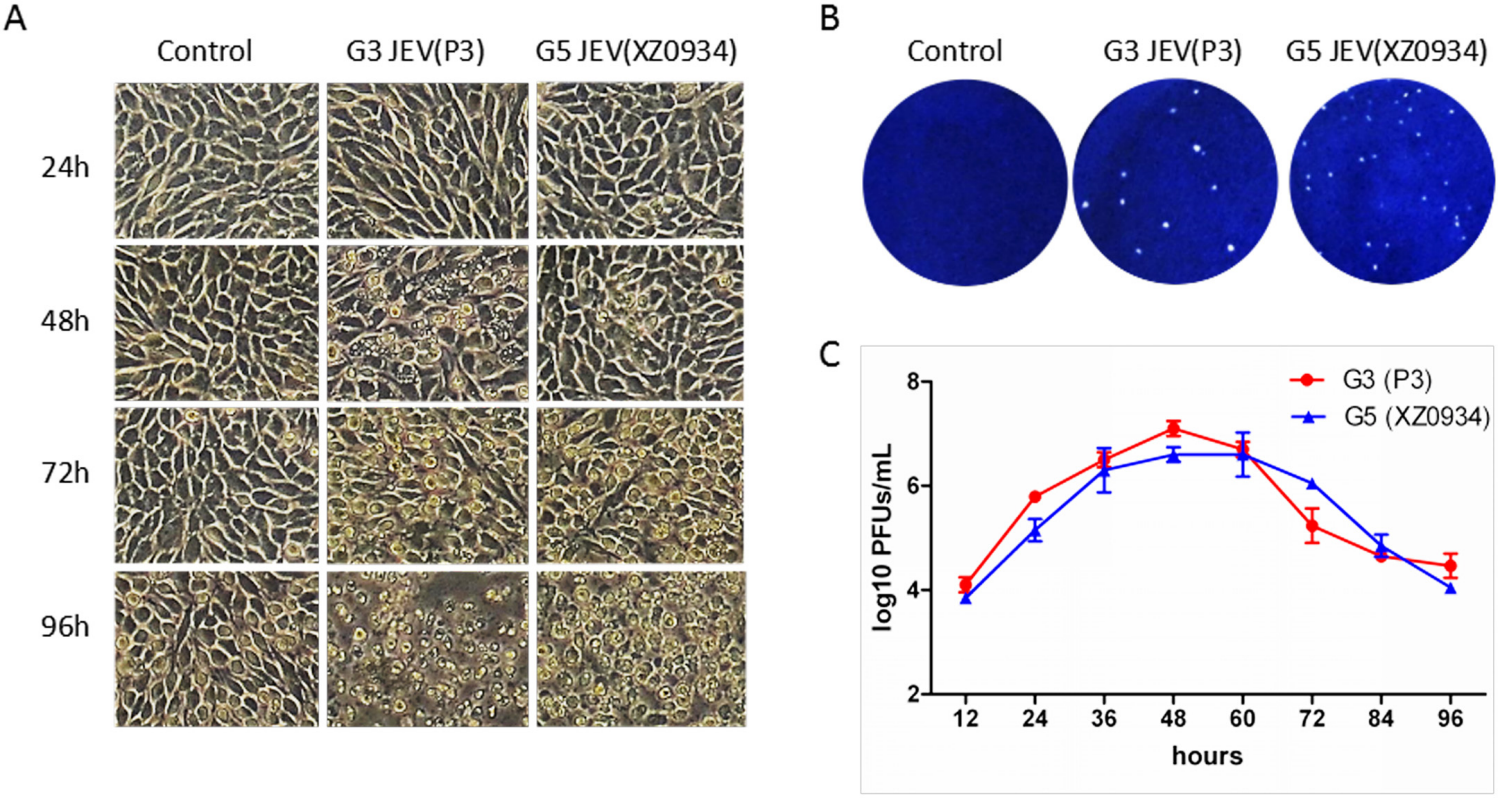
Phenotype comparison between G3 and G5 of JEV. (A) CPE of BHK-21 cells caused by P3 and XZ0934 strains at different times, included rounding, and cell rupture. (B) Plaque phenotype of the JEV P3 and XZ0934 strains in BHK-21 cells after 3 days of infection. (C) Relative multiplication characteristics of the P3 and XZ0934 strains in BHK-21 cells. Cells were plated into 6-well culture plates and infected with the JEV strains at a MOI of 0.01pfu /cell. Values represent the mean and standard deviation from three independent experiments.

CPE due to virus infection showed that G5 JEV (XZ0934 strain) and G3 JEV (P3 strain) can cause BHK cells to shrink and shed (Fig 1A). The plaque diameter of G5 JEV was smaller than that of G3 JEV (Fig 1B). Virus infectivity generated from BHK-21 cells inoculated with MOI of 0.01 showed that G3 JEV and G5 JEV proliferated and reached their highest titers in 48 h and 60 h, respectively (Table 1 and Fig 1C), indicating that the two viruses had substantially similar multiplication capacities in BHK-21 cells.

**Table 1:** Phenotypic characteristics of G5 and G3 JEV.

		G3 JEV (P3)	G5 JEV (XZ0934)
Time of CPE(h)		48–72	48–72
Plaque diameter (mm, n = 8, 3d)		1~1.5(1.23±0.22)	0.5~0.8(0.6±0.14)
Highest titer (pfu/mL)		10^7.1^(48h)	10^6.6^(60h)
Time of death (d)^a^		5	5
Log LD_50_ (/0.03mL)^b^		4.1	3.5
PRNT_90_ titer^c^	G3 JEV antibody	>1:320	1:10
	G5 JEV antibody	1:10	1:40
% Survival (No. of deaths/10 in group) after immunization with LAV/SA14-14-2 JEV vaccine^d^	2510pfu	100% (0/10)	50% (5/10)
	251pfu	100% (0/10)	50% (5/10)
	25pfu	60% (4/10)	50% (5/10)
% Survival (No. of deaths/10 in group) after immunization with inactived P3 JEV vaccine^d^	1:5 dilution	100% (0/10)	80% (2/10)
	1:25dilution	100% (0/10)	80% (2/10)
	1:125dilution	100% (0/10)	60% (4/10)

^a^ Groups of 12 BALB/c neonatal mice were i.c. inoculated with 1×10^3^ pfu / each of Japanese encephalitis virus (JEV) P3 or XZ0934 strains.

^b^ Groups of 5 5~6-week-old BALB/c adult mice were i.p. inoculated with 10-fold diluted JEV P3 or XZ0934 strains, and the incidence and mortality of mice were recorded every day for 21 days. The virulence of each JEV was assessed through calculating 50% lethal dose (LD_50_) using the Reed-Muench method.

^c^ Groups of 7 4-week-old BALB/c mice were separately immunized with thermally inactivated JEV P3 or XZ0934 strains, and the degree of cross-immunoreaction between the two JEV genotypes was measured using plaque reduction neutralization test (PRNT_90_).

^d^ Mice were challenged i.p. with a dose of 500 LD_50_ JEV of P3 or XZ0934 strain 2 weeks after immunization with SA14-14-2 or P3 JEV vaccine, and then monitored daily for 21 days. The survival rate was determined as 100×(numbers challenged—number of deaths)/(numbers challenged).

The 1 ~ 2-day-old BALB/c neonatal mice i.c. inoculated with the two JEV genotypes (10^3^ pfu/mouse; 12/group) all died in the first 5 days. The 5 ~ 6-week-old BALB/c adult mice were i.p. inoculated with the two JEV genotypes (5/group) and the LD_50_ of each virus was calculated to assess the virulence of JEV. The LD_50_ values of the G5 and G3 virus titers (logarithmic) were 3.5 and 4.1, respectively, demonstrating no significant difference in virulence (Table 1).

### Cross-immunoreaction between G3 and G5 JEV

To compare the relative immunogenicities between G3 and G5 JEV, we measured the degree of cross-immunoreaction between the two JEV genotypes using PRNT. The results showed that the PRNT_90_ titer of G3 JEV immune serum against G3 JEV was higher than 1:320 and only 1:10 against G5 JEV. The PRNT_90_ titer of G5 JEV immune serum against G5 JEV was 1:40, and 1:10 against G3 JEV (Table 1). Both JEV genotypes showed strong immunoreactions against the homologous JEV genotype, but lower cross-immunoreactions against the heterologous JEV genotypes.

### Protective efficacy of the current JE vaccine against G5 JEV challenge

**Fig 2:**
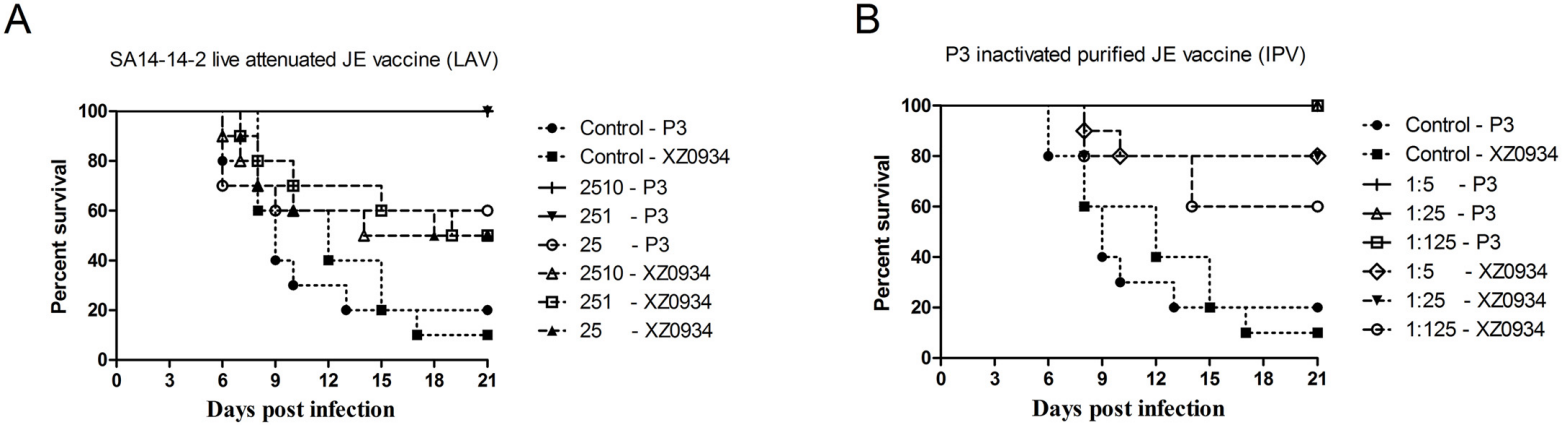
Protection against i.p. challenge with different JE virus strains. Groups of 10 4-week-old mice were immunized with either (A) 10^−3^ (2510 pfu), 10^−4^ (251 pfu) or 10^−5^ (25 pfu) live attenuated vaccine (LAV) or with (B) 1:5, 1:25 or 1:125 dilutions of inactivated purified vaccine (IPV). Then all mice were challenged i.p. with 500 LD_50_ JEV P3 or XZ0934 strains.

Mice were i.p. injected with the JE vaccine at high, medium or low dilution LAV/SA14-14-2 at 2510 pfu, 251 pfu and 25 pfu, IPV/P3 at 1:5, 1:25 and 1:125, respectively. After 14 days from initial immunization, they were then challenged i.p. with 500 LD_50_ of G5 or G3 JEV. The results showed that high and medium doses of LAV completely protected against G3 JEV challenge, but high, medium and low doses of LAV provided only a 50% protection rate against G5 JEV challenge. All IPV doses completely protected against challenge with G3 JEV, while protection rates against challenge with G5 JEV were 80% for high and medium doses in the IPV group (1:5 and 1:25 dilution, respectively) and 60% in the low dose group (1:125 dilution; Table 1 and Fig 2).

### Detection of protective antibodies against G5 JEV in populations vaccinated with the current JE vaccine

**Fig 3:**
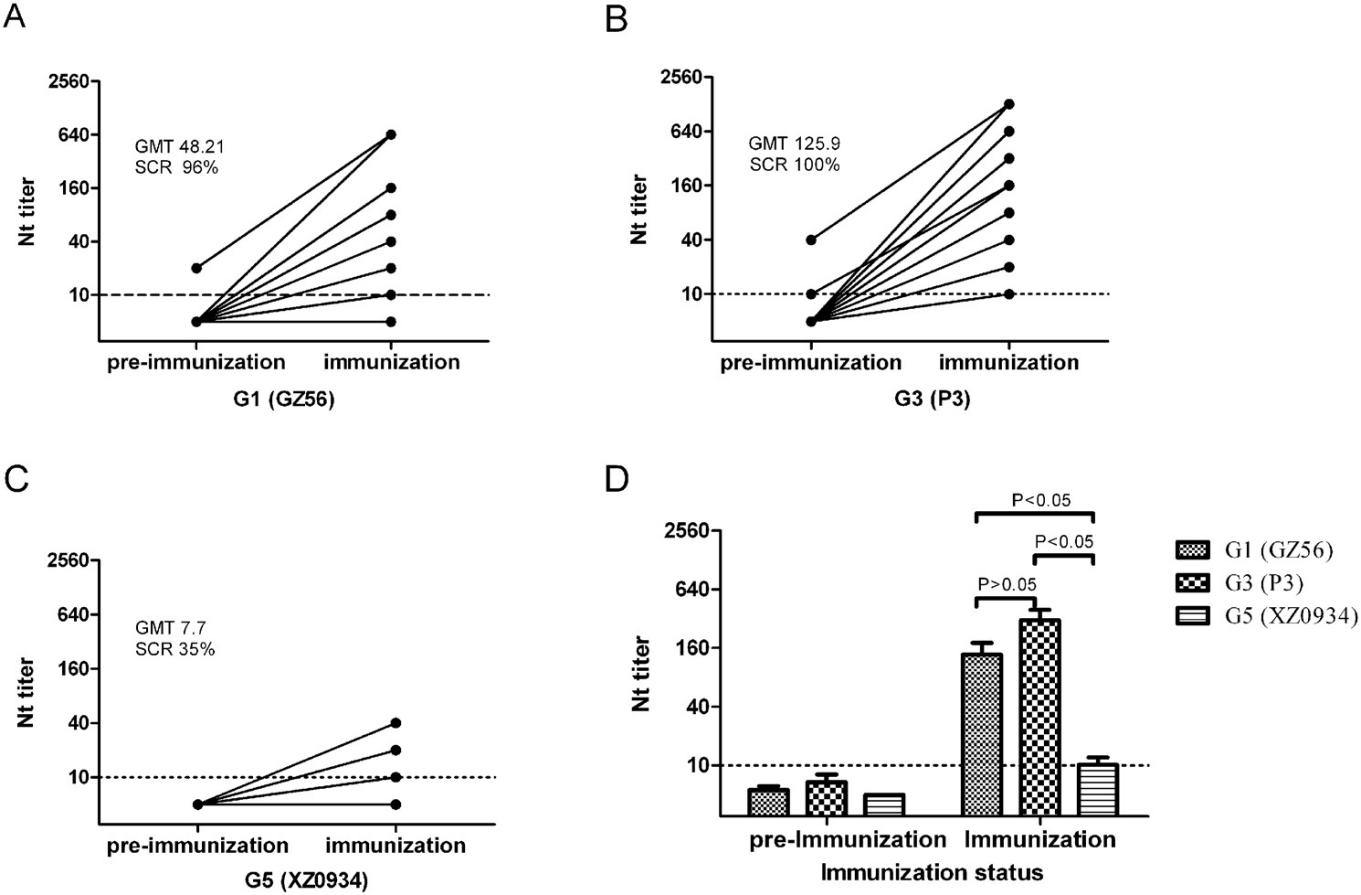
SA14-14-2 JE LAV–induced immune response in two-year-old children 28 days before and after vaccination. PRNT_90_ titers against viral strains of different JEV genotypes are shown before and 28 days after a vaccination: (A) G1 JEV (GZ56 strain), (B) G3 JEV (P3 strain) and (C) G5 JEV (XZ0934 strain). (D) end-point PRNT_90_ titers against three genotypes JEV antibodies were assayed. The gray lines indicate PRNT_90_ titer = 1:10. PRNT_90_ titers of ≥1:10 were considered protective. The seroconversion rates (SCRs) and geometric mean titers (GMTs) are given in each panel. P < 0.05 showed statistically significant.

In this study we compared neutralizing antibody titers against G1, G3 and G5 JEV of 26 pairs of serum samples before and after LAV (SA14-14-2) vaccination. Among the 26 serum samples collected before immunization, one showed 1:10 neutralizing antibody titer against the G1 JEV strain and two showed 1:10 against the G3 JEV strain but all were negative against the G5 JEV strain. After LAV vaccination, neutralizing antibodies against G3 JEV showed 100% positive seroconversion (GMT = 125.9), with the highest neutralizing antibody titer reaching 1:1280 (Fig 3A, S1 Table) and the seroconversion rate (SCR; only one sample <1:10) was 96% against G1 JEV (GMT = 48.21), with the highest neutralizing antibody titer reaching 1:640 (Fig 3B, S1 Table). However, only 35% (9/26) SCR (9 samples ≥1:10) was observed against G5 JEV (GMT = 7.7), including four samples reaching 1:10, three samples reaching 1:20 and two samples reaching a peak antibody titer of 1:40 (Fig 3C, S1 Table).

In serum samples after immunization with the current JE vaccine, neutralizing antibody titers estimated using PRNT_90_ (expressed as GMTs) against G3 JEV and SCRs were analyzed and the results showed that when neutralizing antibodies against G3 JEV serum were ≥ 1:40, SCRs against G1 JEV were as high as 100%, but none were seropositive against G5 JEV (0% SCRs). When neutralizing antibodies against G3 JEV serum were 1:160–1:320, SCRs against G5 JEV were only 50%. Protective antibodies were produced against G5 JEV (SCRs up to 100%) only when the dilution of G3 JEV serum was higher than 1:320. The results are shown in Table 2.

**Table 2:** Strain-specific protection threshold of Japanese encephalitis virus vaccine.

PRNT_90_ against G3(P3)	Sample size	PRNT_90_ GMT against JEVs(95% CI)			Seropositivity rate against JEVs(%)^a^		
		G1 (GZ56)	G3 (P3)	G5 (XZ0934)	G1 (GZ56)	G3 (P3)	G5 (XZ0934)
10–20	4	**8.41(4.85–14.6)**	16.82(9.69–29.19)	**5(5–5)**	75	100	0
40–80	9	23.33(14.97–36.37)	63.5(48.65–82.88)	**5(5–5)**	100	100	0
160–320	8	61.69(40.08–94.95)	190.3(145.5–248.8)	**7.07(5.19–9.64)**	100	100	50
>320	5	485(224.6–1047)	1114(758.4–1637)	26.39(16.47–42.28)	100	100	100
JEV Overall GMT	26	48.21(27.15–85.6)	125.9(71.92–220.3)	7.7(5.82–10.09)	96	100	35

^a^ Defined as plaque reduction neutralization test (PRNT_90_)≧1:10 using BHK-21 cells

Boldface indicates titers negative as PRNT_90_<1:10 and is considered below the protective threshold

### Detection of neutralizing antibody against G5 JEV in serum samples collected during acute phase from clinical JE patients

This study further examined levels of neutralizing antibody against G5 JEV in serum samples from clinically diagnosed JE patients. All 45 serum samples were confirmed IgM-positive when using two JEV IgM antibody detection kits. Neutralizing antibodies against G1, G3 and G5 JEV were detected in all 45 serum samples. The results showed that neutralizing antibodies (≥ 1:10–1280) against G3 JEV were detected in all serum samples from JE patients with serum GMT of 43.2. Neutralizing antibodies (≥ 1:10–80) against G1 JEV were detected in 87% (39/45) of serum samples with serum GMT of 16.37. Additionally, neutralizing antibodies against G5 JEV were detected in only 64% (29/45) of serum samples with serum GMT of 11.14. Evaluation of JE patient’s age and neutralizing antibodies against G5 virus showed that 41% (9/22) of JE patients 1–15 years of age and 87% (20/23) of patients over 16 years of age had positive neutralizing antibodies against G5 JEV (Table 3).

**Table 3:** The neutralizing antibody response against different genotype JEV in serum of acute phase from clinical JE patients in different ages.

Samples		JEV PRNT90 GMT (95% CI)			PRNT90≥10 ^a^		
					No. of cases		
					(% in this age group)		
Ages (Years)	No. of cases	G1 (GZ56)	G3 (P3)	G5 (XZ0934)	G1 (GZ56)	G3 (P3)	G5 (XZ0934)
1–15	22	10.32 (8.27–12.89)	22.69 (15.59–33.01)	7.53 (5.9–9.62)	17 (77%)	22 (100%)	9 (41%)
16–40	5	52.78 (9.86–282.5)	160 (21.27–1024)	20 (5.13–77.97)	5 (100%)	5 (100%)	4 (80%)
>41	18	20.79 (13.69–31.56)	65.99 (37.99–114.6)	14.7 (10.26–21.05)	17 (94%)	18 (100%)	16 (89%)
Total	45	16.37 (12.42–21.58)	43.2 (29.77–62.69)	11.14(8.81–14.09)	39 (87%)	4 (100%)	29 (64%)

^a^ Defined as plaque reduction neutralization test (PRNT_90_)≧1:10 using BHK-21 cells

## Discussion

### Poor cross-immunogenicity

G5 JEV and G3 JEV wild-type isolates were used in this study. Comparative studies of wild strains showed that the biological characteristics of G5 JEV, such as CPE, virus titers and virus lethality to animals, were similar to those of G3 JEV. However, cross-neutralization studies showed that antigenic cross-reactivity between G3 and G5 JEV was low (Table 1) and this was also reflected in the low cross-protectivity studies. A study of a G5 JEV infectious molecular clone (the infectious molecular clone was prepared from the full-length cDNA of G5 JEV XZ0934 strain) showed similar results [20] as did the use of G5 JEV wild strains isolated from viral JE patients in 1951 [14]. Further animal experiments also showed that the protection capacity of the current JE vaccine against G5 JEV challenge on animals was only 50% (Table 1). These results indicate that G3 and G5 JEV were significantly different in terms of their capacity to stimulate cross-protective immune responses to the heterologous virus. Our present study about molecular characterization of full-length genome of G5 JEV (SA14-14-2 strain) shows that G5 JEV is significantly different from that of the known G3 JEV (SA14-14-1) [12]. The open reading frame (ORF) of G5 JEV(10302 nucleotides (nt)) was 3 nt longer than that of G3 JEV(10299 nt), which encoding an additional amino acid (aa) Ser residue in the NS4A gene of G5 JEV. What is more, They share low identity of 90.7% in nt and 79.3% in aa. The envelope protein (E protein) encoded by the E gene plays an important role in eliciting protective neutralizing antibodies and is crucial for the neurovirulence of JEV. Except the similar 8 key aa residues in E protein for virulence of JEV, the aa identity of E protein between G5 and G3 JEV is only 89.6%. These heterologous differences may involve in the induction of genotype-specific neutralizing antibodies and led to poor cross-immunogenicity between heterologous G3 and G5 JEV.

### Low protective efficacy of the current JE vaccine against G5 JEV

To date, regardless of whether attenuated or inactivated JEV vaccines are used, they are all currently based on JEV strains representing the single genotype 3 (G3) [21]. Previous studies showed these vaccines can protect against G1–G4 JE virus infection [8,18,19]. In this study, PRNT_90_ assays were used to detect neutralizing antibodies against JE viruses in specimens collected from JE-vaccinated healthy children. The results showed that after receiving JE vaccines, SCRs of neutralizing antibodies against G3, G1 and G5 JEV in the serum samples were 100%, 96% and 35%, respectively (Fig 3). SCRs of neutralizing antibodies against G3 and G1 JEV in this study were similar to those observed in previous research; G3 was 100% and G1 slightly lower at 97% (45/47) [8, 18, 19]. However, in this study, neutralizing antibodies against G5 JEV were detected in only 35% of the healthy children after they received the JE vaccination and 100% SCRs against G5 were detected only when the titer of neutralizing antibodies against G3 JEV was as high as 1: 320 (Table 2). This study is the first to report the low SCR of G5 JEV after administration of the current JE vaccine. Although a larger number of post-vaccination serum samples needs to be evaluated, the results of this study indicate that the protective efficacy of the current JE vaccine is very limited against G5 JE virus infection, regardless of the SCRs or protective neutralizing antibody titers.

### Patients with natural infectious JE have a risk of re-infection with G5 virus

In this study, JEV IgM-positive serum samples (n = 45) collected from clinically diagnosed JE patients were used to evaluate them for the presence of G5 JEV-specific neutralizing antibodies. The positive rates of neutralizing antibodies against G3 and G1 JEV in the serum were 100% (45/45) and 89% (39/45), respectively. However, the positive rate of neutralizing antibodies against G5 JEV was only 64% (29/45). Further analysis showed that the positive rates of neutralizing antibodies against G5 JEV in JE patients 1–15 years of age were 41% (9/22) and 87% (20/23) in JE patients over 15 years of age, indicating that JE patients of different ages showed different levels of G5 JEV protective antibodies. This possibly reflects the greater number of exposures to JEV in the older age-group. Additionally, in this study, the serum sample of a 9-year-old child collected on the 26th day after onset of JE (S2 Table, the 1st case) and serum samples of two children collected on the 11th day after onset of JE (S2 Table, the 5th and 6th cases), showed detectable seroconversion antibodies against G3 JEV (PRNT_90_≥1:10). However, antibodies against G5 were all negative (PRNT_90_<1:10). These results indicate that within 11–26 days after being infected with JEV, patients failed to produce protective antibodies against G5 JEV. Therefore, pediatric patients with natural JEV infection still had significant potential risk of G5 virus re-infection. Based on our results, whether patients infected with JEV can produce protective antibodies against G5 JEV in the recovery period was inconclusive. The results did, however, imply that JE pediatric patients may not produce protective antibodies against G5 JEV after infection with non-G5 strains of JEV.

### Significance to public health

JE is a vaccine-preventable disease and widespread and long-standing vaccination programmes have significantly reduced JE cases in traditional endemic areas such as Japan and Korea. However, since the discovery of the G5 virus in South Korea in 2009 [11], JE cases in Korea have been reported yearly (26 in 2010, 3 in 2011, 20 in 2012, 14 in 2013 and 26 in 2014) [22]. Moreover, six G5 JEVs have been recently detected in local Korean *orientalis* and *Culex pipiens* mosquitoes, suggesting that, in addition to the dominant *Culex tritaeniorhynchus*, other species of mosquito can spread G5 JEV in Korea [23]. The Chinese government included the JE vaccine in their EPI management programme in 2008, allowing school-age children to receive the JE vaccination for free. However, thousands of JE cases still occurred yearly after 2008 [24, 25]. Therefore, research on whether JE patients in Korea or China were related to the emerging G5 JEV infection needs to be carried out. To evaluate in detail the protective efficacy of the current JE vaccine against G5 JEV infection, increasing the surveillance of G5 JEV in vectors in endemic areas, detecting levels of G5 JEV protective antibodies across different age groups in JE-endemic areas, and finding clinical JE cases due to G5 JEV infection should be implemented.

## Supporting Information

S1 Table Neutralizing antibody titers against different JEV strains in vaccines (children) with the live vaccine SA14-14-2. 26 serum samples from JE-vaccinated children were collected from two-year-old children 28 days before and after vaccination SA14-14-2 JE live attenuated vaccine (LAV), (Wuhan Institute of Biological Products Co. Ltd Liability, Wuhan, China). The PRNT_90_ method was used to detect neutralizing antibodies against different genotypes (G1, G3 and G5) of JEV in these subjects.
(XLS)

S2 Table Neutralizing antibody titers against different JEV strains in serum of acute phase from clinical JE patients. 45 serum samples were collected from patients diagnosed with JE and two kits of Capture ELISA were used to duplicate test these serum samples for confirmed JE patients. Then The PRNT_90_ method was used to detect neutralizing antibodies against different genotypes (G1, G3 and G5) of JEV in these serum samples.
(XLS)
